# Reduced levels of circulating progenitor cells in juvenile idiopathic arthritis are counteracted by anti TNF-α therapy

**DOI:** 10.1186/s12891-015-0555-9

**Published:** 2015-04-30

**Authors:** Giorgia Martini, Francesca Biscaro, Elisa Boscaro, Fiorella Calabrese, Francesca Lunardi, Monica Facco, Carlo Agostini, Francesco Zulian, Gian Paolo Fadini

**Affiliations:** Paediatric Rheumatology Unit, Department of Paediatrics, 35128 Padova, Italy; Department of Medicine, University of Padova, Via Giustiniani, 2, 35128 Padova, Italy; Department of Cardiovascular and Thoracic Sciences, University of Padova, Via Giustiniani, 2, 35128 Padova, Italy

**Keywords:** Stem cells, Cardiovascular, Endothelium, Angiogenesis

## Abstract

**Background:**

Endothelial progenitor cells (EPC) promote angiogenesis and vascular repair. Though reduced EPC levels have been shown in rheumatoid arthritis, no study has so far evaluated EPCs in children with juvenile idiopathic arthritis (JIA). We aimed to study circulating EPCs in children with JIA, their relation to disease activity, and effects of anti TNF-α treatment.

**Methods:**

Circulating EPCs were quantified by flow cytometry based on CD34, CD133 and KDR expression in peripheral blood of 22 patients with oligoarticular JIA and 29 age-matched controls. EPCs were re-assessed in children with methotrexate-resistant oligo-extended JIA before and up to 12 month after initiation of anti-TNF-alpha therapy. Plasma concentrations of inflammatory and EPC-regulating factors were measured using a multiplex array. Confocal immunofluorescence was used to demonstrate EPCs in synovial tissues.

**Results:**

Children with active JIA showed a significant reduction of relative and absolute counts of circulating progenitor cells and EPCs compared to age-matched healthy controls. CD34^+^ cell levels were modestly and inversely correlated to disease activity. A strong inverse correlation was found between serum TNF-α and EPC levels. In 8 patients treated with anti TNF-α agents, the number of EPCs rose to values similar to healthy controls. CD34^+^KDR^+^ EPCs were found in the synovial tissue of JIA children, but not in control.

**Conclusions:**

Children with JIA have reduced levels of the vasculoprotective and proangiogenic EPCs. While EPCs may contribute to synovial tissue remodelling, EPC pauperization may indicate an excess cardiovascular risk if projected later in life.

## Background

Juvenile Idiopathic Arthritis (JIA) is a heterogeneous condition including all forms of chronic arthritis of unknown origin with onset before the age of 16 and is currently divided into different subgroups according to the clinical features at onset [1]. JIA is characterised by chronic inflammation of single or multiple joints leading to synovial tissue hyperplasia, cartilage destruction and bone erosion [2]. This process results from the proliferation of synoviocytes and the recruitment of activated inflammatory cells from the peripheral circulation [3,4]. One important aspect of synovial hyperplasia is the formation of new blood vessels, or angiogenesis, which increases the homing capacity of inflammatory cells to the synovial tissue and fluid [4-6].

Endothelial progenitor cells (EPCs) are bone marrow (BM) derived cells involved in the processes of angiogenesis and endothelial repair [7]. EPCs are part of a larger population of circulating progenitor cells (CPCs), such as the hematopoietic CD34^+^ population, which are *per se* involved in angiogenesis [8]. Circulating EPCs and CPCs are reduced in virtually all clinical conditions associated with increased cardiovascular risk, such that their levels in the bloodstream are now considered as independent biomarkers and inverse predictors of future cardiovascular disease [7]. EPCs are also affected by inflammatory diseases, with a possible early rise driven by acute inflammation, followed by absolute depletion in the chronic phases [9]. Several reports indicate that EPCs and CPCs are quantitatively and qualitatively affected in adults with rheumatoid arthritis (RA) [10], systemic lupus erythematosus [11] and systemic sclerosis [12], possibly reflecting impaired angiogenesis and/or increased cardiovascular risk. However, to the best of our knowledge, no study has so far analysed EPCs and CPCs in the setting of JIA.

Therefore, aim of this study was to evaluate the circulating levels of CPC and EPC in children with JIA, examine whether they correlate with clinical data, and whether they are influenced by medical treatment, in particular with anti TNF-α agents.

## Methods

### Patients

Peripheral blood samples from consecutive patients fulfilling the revised criteria for JIA, according to the ILAR (International League of Associations for Rheumatology) Durban criteria [1] and managed at the Paediatric Rheumatology Unit of Padova University were studied. Peripheral blood samples were drawn for the determination of EPCs, CPCs, inflammatory markers and progenitor cell mobilizing factors. At the time of blood sample collection, all included patients had persistently active disease: some of them were undergoing intraarticular corticosteroid injection, other patients with methotrexate (MTX)-resistant oligo-extended JIA were starting anti-TNF-α treatment. In these patients, blood samples were collected 3 and 6 months after initiation of the anti TNF-α agent.

The following clinical data were collected: age at onset of JIA, disease duration, concomitant medications, overall assessment of disease activity by the physician’s visual analogue scale (VAS) (range 0–100 mm), number of active joints (joints with swelling not caused by deformity, or joints with limited motion, and with pain, tenderness, or both), laboratory markers of inflammation, including erythrocyte sedimentation rate (ESR) and C-reactive protein (CRP) with normal value up to 25 mm/h and up to 6 mg/L, respectively, according to our laboratory standard.

Age- and sex-matched healthy subjects were recruited from the service of Laboratory Medicine used as controls. The study was approved by the University Hospital of Padova and consent was obtained from the parents of all the children taking part in this study.

### Quantification of EPC in peripheral blood by flow cytometry

Progenitor cells in whole peripheral blood were quantified based on the expression of surface antigens with direct 3-color analysis, as described before [13], using fluorescein isothiocyanate (FITC)-conjugated, phycoerythrin (PE)-conjugated and allophycocyanin (APC)-conjugated monoclonal antibodies (mAbs) by flow cytometry (FACSCalibur; Becton, Dickinson and Company, Franklin Lakes, NJ, USA). Briefly, before staining with specific monoclonal antibodies, cells were treated with fetal calf serum for 10 minutes and then the samples were washed with buffer containing phosphate-buffered saline and 0.5% bovine albumin. Then, 150 μl of APC-conjugated anti-human CD133 mAb (Miltenyi Biotec, Bergisch Gladbach, Germany) and 10 μl of PE-conjugated anti-human KDR mAb (R&D Systems Inc., Minneapolis) followed by incubation at 4°C for 30 minutes. Unlabeled cells or anti-isotype antibody served as a control. The frequency of peripheral blood cells positive for the above reagents was determined by a two-dimensional side scattered-fluorescence dot plot analysis of the samples, after appropriate gating. After morphological gating to exclude granulocyte and cell debris, we gated CD34^+^ peripheral blood cells and then examined the resulting population for dual and triple expression of KDR and CD133. CPCs were defined as CD34^+^, CD133^+^ or CD34^+^CD133^+^ cells. Circulating EPCs were defined as CD34^+^KDR^+^, CD133^+^KDR^+^ or CD133^+^CD34^+^KDR^+^ cells. For each analysis, 5 x 10^5^ cells were acquired and scored using a FACS-calibur analyzer (Becton, Dickinson and Company). Data were processed using the Macintosh CELLQuest software program (Becton, Dickinson and Company). The same trained operator, who was blind to the clinical status of the patients, performed all the tests. Data are expressed as cells/10^6^ events or as cells/ml, by multiplying by white blood cell count.

### Determination of inflammatory mediators and progenitor cell mobilising factors

Plasma concentrations of IL-1β, IL-1ra, IL-6, bFGF, G-CSF, PDGF-bb, TNF-α and VEGF were measured using a customized multiplex suspension array (BioRad Laboratories, Milan, Italy) according to the manufacturer’s instructions. This assay allows the simultaneous detection of multiple molecules in a single well on 96-well microplate, using specific antibody-coupled fluorescently dyed beads in a sandwich immunoassay. White blood cells, ESR and CRP were determined on an automated modular Roche Cobas Analyzer.

### Confocal microscopy

Synovial tissue was obtained at time of arthroscopic synovectomy in 3 patients with oligoarticular-onset JIA and in 1 subject with non-inflammatory disease (hypermobile joint), after informed consent was obtained from the parents. In JIA, the procedure was performed for clinical indication in the presence of persistently inflamed joints, not responding either to systemic anti-inflammatory therapy or to intraarticular corticosteroid injections. Confocal microscopy was used to confirm the co-expression of CD34 and KDR (VEGFR2) in synovial tissue cells, indicating locally homed EPCs. Paraffin sections were prepared for immunofluorescent labelling. Briefly, primary antibodies against CD34 (ready to use, Delta) and VEGFR2 (1:200 Santa Cruz) and secondary antibodies (goat anti-mouse IgG and goat anti-mouse IgG) conjugated with Cy3 and Cy2 (Sigma) were used, respectively. TO-PRO 3 far red fluorescent probe (Molecular Probes, Invitrogen) was used to label nuclei. Double labelling using both antibodies on the same section was performed. Primary and secondary antibodies were incubated for 1 h at room temperature. Slides were stored at 4°C and analysed within 24 h. As a negative control, the primary antibody was omitted. Immunofluorescence was evaluated with a confocal microscopy (Leica TCS SL, Heidelberg, Germany) by using 488, 543 and 642 nm excitation. Images were analyzed using Adobe Photoshop 7.0.

### Statistical analysis

Normal distribution of variables was assessed using the Kolmogorov-Smirnov test. Normal variables are expressed as mean ± standard error, while non-normal variables are expressed as median (interquartile range, IQR). Differences between normal continuous variables were assessed using unpaired two-tail Student’s t test, while the Mann–Whitney test was used for non normal continuous variables. The chi square test was used to compare categorical variables between two groups. Differences in variables assessed before and after anti-TNF therapy were assayed using paired two-tail Student’s t test the Wilkoxon rank test. Linear correlations were evaluated by Pearson’s r coefficient. Statistical significance was accepted at p < 0.05.

## Results

Twenty-two JIA patients were included in the study, all having a history of oligoarticular onset, with 9 showing persistently oligoarticular course and 13 with extension to polyarticular course (oligo-extended JIA). At the time of blood sampling, 14 patients underwent intraarticular steroid injection and 8 patients with MTX-resistant disease were started on anti TNF-α agents (6 etanercept and 2 infliximab). The clinical characteristics of the patients are summarized in Table 1. Twenty nine control subjects were included and were aged 8.6 ± 1.0 years (range 2–15 years), 44% males, and were free from any acute and chronic disease.

**Table 1:** **Clinical characteristics of patients included in the study**

**Clinical characteristic**	**Oligo-extended**	**Oligoarticular**
**No.**	13	9
**Age mean (range), years**	10.0 (3.0-21.6)	8.0 (4.4-14.3)
**Disease duration, years**	5.8 (0.4-17.4)	2.1 (0.2-13.3)*
**ESR mean (range), mm/h**	39.8 (10–101)	35.6 (14.0-90.0)
**CRP mean (range), mg/L**	23.5 (3.3-72.0)	9.4 (3.0-30.6)*
**VAS mean (range), mm**	7.5 (2.0-9.7)	7.2 (4.0-9.0)
**No. of active joints**	3.9 (1–13)	2.3 (1–5)*
**Previous treatment**	None (3 pts)	
Methotrexate (MTX, 4 pts)		
Naproxen (NPX, 2 pts)		
MTX/NPX (3 pts)		
MTX/prednison (2 pts)	None (3 pts)	
Naproxen (6 pts)		

*p < 0.05.

**Figure 1:**
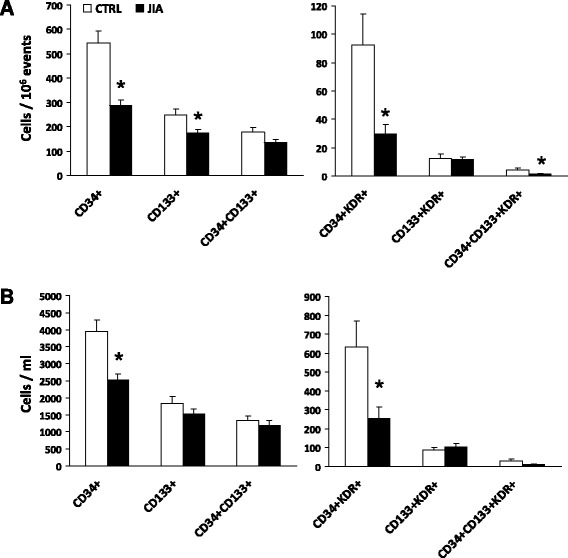
Progenitor cell phenotypes in JIA compared to control (CTRL) children. Cell counts are expressed, on different scales, as relative to one million total cytometric events in **(A)** and as absolute concentration in **(B)**. *p < 0.05 versus CTRL subjects. Generic CPCs include CD34^+^, CD133^+^, CD34^+^CD133^+^ cells, whereas endothelial committed progenitor cells (EPCs) include CD34^+^KDR^+^, CD133^+^KDR^+^, CD34^+^CD133^+^KDR^+^ cells.

Children with active JIA showed significantly lower levels of multiple CPC and EPC subtypes in peripheral blood compared to age-matched healthy controls. Specifically, significant reductions in relative cell counts were detected for the total CD34^+^ cell population (−47%), CD133^+^ cells (−30%), CD34^+^KDR^+^ cells (−68%) and CD34^+^133^+^KDR^+^ (−71%), while there were non-significant trend reductions in CD34^+^CD133^+^ and CD133^+^KDR^+^ cells (Figure 1A). Absolute CD34^+^ and CD34^+^KDR^+^ cell counts were also reduced in JIA as compared to control subjects (Figure 1B).

**Figure 2:**
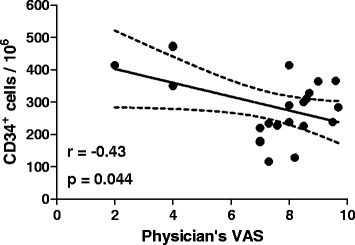
Correlation between CD34^+^ cells and disease activity. A significant inverse linear correlation was detected between total CD34^+^ cells and the physician’s visual analogue scale.

Among all clinical characteristics, we only found a significant negative correlation between CD34^+^ cell levels and the physician’s visual analogue scale (Figure 2), though this was mainly attributable to 3 patients with low disease activity. There was no association between progenitor cells and the clinical subtype (persistent oligoarticular or extended oligoarticular), disease duration, and ongoing treatment regimens with NSAIDs and MTX.

JIA children, compared to controls, showed significantly higher concentrations of plasma TNF-α (95.9 ± 46.1 versus 22.7 ± 3.8 pg/mL; p = 0.002) and IL-6 (19.3 ± 3.2 versus 11.8 ± 1.8 pg/mL; p = 0.04), while there were no differences in IL-1β, IL-1ra and progenitor cell mobilizing factors (bFGF, G-CSF, PDGF-bb, VEGF and SCF). Within the JIA group, there were negative correlations between TNF-α levels and the CD34^+^KDR^+^ (r = −0.79; p < 0.001) and CD34^+^133^+^KDR^+^ (r = −0.52; p = 0.004) EPC phenotypes.

**Figure 3:**
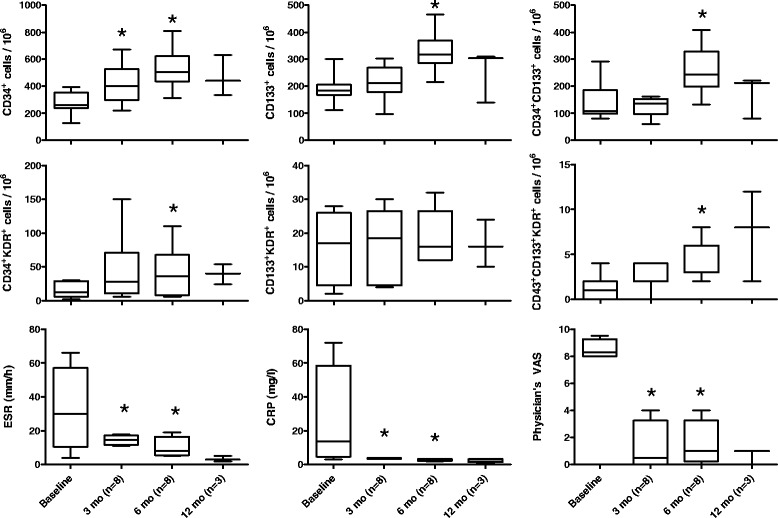
Effects of anti-TNF-alpha therapy on the levels of progenitor cell phenotypes and clinical severity. ESR, CRP, VAS and progenitor cell phenotypes were measured at baseline and after 3 and 6 months in 8 children with persistently active JIA. In 3 individuals, a blood sample was also performed at 12 months. *p < 0.05 versus baseline.

In a group of 8 children with persistently active disease despite treatment with MTX, the initiation of anti TNF-α agents was followed by a significant increase in CPC and EPC, at both 3 and 6 months after the start of treatment. Consistently, we found significant clinical improvements in terms of ESR, CRP, VAS and number of active joints (Figure 3). In 3 subjects, who underwent a blood sampling at 12 months, the increase in EPC and CPC appeared to be sustained over time.

**Figure 4:**
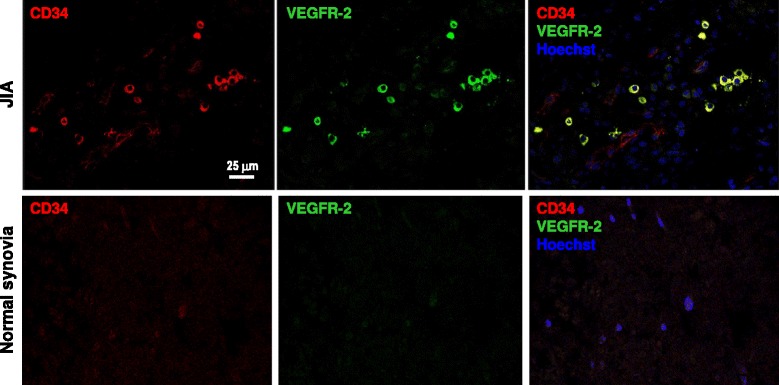
Bona fide EPC labelling in synovial tissue of JIA patients. A representative confocal imaging analysis of synovial tissue from a JIA patient (upper lane) and from a child who underwent surgical debridement for a non-inflammatory condition (lower lane). The staining for CD34 and VEGFR-2 (KDR) shows double positive CD34^+^KDR^+^ cells (bona fide EPCs) in clusters or as isolated cells only in the JIA case, while the control was completely negative. Note that, as expected, CD34 also labels microvessels, although at lower intensity compared to isolated EPCs.

To understand whether EPC might be involved in intra-articular tissue remodelling in the setting of JIA, we performed confocal microscopy imaging of synovial tissue obtained from patients with oligoarticular-onset at time of synoviectomy. In the synovial tissue, CD34 labeled blood vessels and rounded cells in the interstitium, that appear to be either isolated or in clusters. CD34^+^ rounded cells, but not CD34^+^ elongated cells in blood vessels, were also brightly positive for VEGFR2 (KDR). Therefore, these cells can be *bona fide* defined as tissue-resident CD34^+^KDR^+^ EPCs, similar to circulating EPCs. A control non-inflammatory synovial tissue was completely negative for EPC markers (Figure 4).

## Discussion

In this study we show for the first time that, compared to age-matched healthy children, subjects with active JIA displayed a marked reduction of multiple CPC and EPC phenotypes, while such defect was counteracted by anti TNF-α therapy.

Reductions in EPC levels have been previously shown in the setting of adult RA and other rheumatic diseases, including systemic lupus erythematosus, systemic sclerosis, and Behcet disease [9-13]. There are at least two possible explanations for the reduced levels of pro-angiogenic and endothelial-reparative cells in JIA. First, chronic inflammation, by continuously challenging and stressing the bone marrow, may progressively exhaust progenitor cell reserve and/or mobilization capacity [14]. Impaired bone marrow function and pauperization of EPC progenies have been demonstrated to account for circulating EPC reduction in diabetes and cardiovascular disease [15,16], but fewer data are available on bone marrow niche structure and function in rheumatic diseases. Padadaki et al. found reduced bone marrow CD34^+^ cells and increased apoptosis, along with defective stromal cell function, which might account for impaired stem cell mobilization in RA patients [17]. Future studies on this topic may be important to better understand the systems biology of rheumatic diseases, as the bone marrow is being recognized as a central housekeeper of multiple organ and tissue regenerative potential.

Second, the existence of cells with a phenotype corresponding to circulating EPCs (CD34^+^KDR^+^) in the synovial tissue of JIA patients suggests that EPCs can be recruited from the bloodstream to the affected joints and might therefore contribute to tissue remodeling and formation of pannus, which is a highly vascularized pathologic structure. The presence of CD34^+^KDR^+^ cells was previously shown also in the synovial tissue of patients with RA and osteoarthritis [18], indicating that this is not a specific finding in JIA. While the homing hypothesis is supported by preclinical studies showing recruitment of bone marrow-derived cells to sites of inflamed joints in experimental arthritis and by the observation that VEGF blockade in murine collagen-induced arthritis (CIA) significantly reduces synovial inflammation [19], it is unclear to what extent can EPC recruitment to the synovial tissue reduce the steady-state circulating EPC levels. In addition, this mechanism would not be common to other rheumatic diseases characterized by unaffected (e.g. SLE) or even impaired (SS) angiogenesis, yet in which circulating EPCs are similarly reduced.

Whichever the cause of progenitor cell reduction, it is remarkable that this occurs in JIA children at the same extent as in adults with RA [20]. Generic CPC, such as CD34^+^ cells, and EPCs are involved in angiogenesis and endothelial repair [8,21]. Therefore, a chronic shortage of these cells in JIA children may, over the decades, translate into impaired vascular homeostasis and promote cardiovascular disease later in life [22]. The cardiovascular risk associated with RA in adults is well established [23]. In the last years, there is a growing interest in investigating whether children with JIA may also have an increased cardiovascular risk, as inflammation is a common driver in the pathogenesis of both arthritis and atherosclerosis, which starts early in life [24]. For instance, children with JIA have elevated levels of pro-inflammatory adhesion molecules suggestive of endothelial activation and known to be associated with vascular dysfunction [25]. Moreover, several studies exploring vascular function with blood pressure, aortic stiffness and other surrogate markers of CVD, such as carotid-intima media thickness (cIMT) and brachial artery flow-mediated dilatation (FMD), suggest that patients with JIA may have vascular dysfunction [26,27].

On this basis, understanding the mechanisms driving the pathological change in the vascular regenerative capacity in JIA is of paramount importance. Herein, we show that circulating EPC reduction was correlated to increased plasma TNF-α concentrations. In vitro, TNF-α was previously shown to reduce EPC generation and survival, through the p38 mitogen associate protein kinase pathway [28]. The negative role played by TNF-α in reducing EPCs in JIA is substantiated by the finding that EPCs were restored toward normal levels after initiation of anti TNF-α therapy. Similar results have been obtained in studies on adults with RA [20,29], but it should be acknowledged that a definite demonstration that restoration of EPC levels is directly mediated by blocking TNF-α signaling is still missing. Indeed, a generalized improvement in the inflammatory milieu may also favorably affect EPCs, independently of TNF-α. In addition, one limitation this longitudinal observational study is that the treatment was decided on clinical ground and not randomly assigned.

Despite efforts made by several investigators for a standardization of the exact EPC phenotype, no general consensus is available [7]. Even the EUSTAR guidelines do not provide a balanced view, as different aspects, ranging from enrichment strategies to the choice of surface biomarkers, are questionable [30]. While quantification of the CD34^+^KDR^+^ events in the lymphomonocyte gate remains the most accepted way of determining EPC levels, the simultaneous analysis of several generic and endothelial progenitor cell phenotypes can strengthen the results. In fact, our study show that multiple progenitor cell subtypes were reduced in JIA, clearly pointing to a generalized pauperization of regenerative cells.

## Conclusions

We show for the first time that children with JIA have significantly reduced levels of circulating cells involved in endothelial repair and angiogenesis. This novel finding, though observational, provides a clue to the worrisome excess of cardiovascular risk in JIA children, which can impact their life in adulthood. Reversibility of this defect after anti TNF-α therapy is a remarkable observation that needs to be confirmed in controlled trials. The effect on EPCs might be considered as a surrogate to assess the favorable cardiovascular effects of TNF blocking.

## Abbreviations

APC: Allophycocyanin
bFGF: Basic fibroblast growth factor
BM: Bone marrow
CD: Cluster of differentiation
CPC: Circulating progenitor cells
CRP: C-reactive protein
CVD: Cardiovascular disease
EPC: Endothelial progenitor cells
ESR: Erythrocyte sedimentation rate
EUSTAR: Eular scleroderma trial and research
FACS: Fluorescence activated cell sorting
FITC: Fluorescein isothiocyanate
FMD: Flow mediated dilation
G-CSF: Granulocyte colony stimulation factor
IL: Interleukin
ILAR: International League of Associations for Rheumatology
IMT: Intima media thickness
IQR: Interquartile range
JIA: Juvenile idiopathic arthritis
KDR: Kinase insert domanin receptor
MTX: Methotrexate
NSAID: Non steroid anti-inflammatory drug
PDGF: Platelet derived growth factor
PE: Phycoerythrin
RA: Rheumatoid arthritis
TNF: Tumor necrosis factor
VEGFR2: Vascular endothelial growth factor receptor 2

## References

[1] Petty RE, Southwood TR, Baum J, Bhettay E, Glass DN, Manners P (1998). Revision of the proposed classification criteria for juvenile idiopathic arthritis: Durban, 1997. J Rheumatol.

[2] Prakken B, Albani S, Martini A (2011). Juvenile idiopathic arthritis. Lancet.

[3] Firestein GS (1999). Starving the synovium: angiogenesis and inflammation in rheumatoid arthritis. J Clin Invest.

[4] Sullivan KE (2007). Inflammation in juvenile idiopathic arthritis. Rheum Dis Clin North Am.

[5] Scola MP, Imagawa T, Boivin GP, Giannini EH, Glass DN, Hirsch R (2001). Expression of angiogenic factors in juvenile rheumatoid arthritis: correlation with revascularization of human synovium engrafted into SCID mice. Arthritis Rheum.

[6] de Jager W, Hoppenreijs EP, Wulffraat NM, Wedderburn LR, Kuis W, Prakken BJ (2007). Blood and synovial fluid cytokine signatures in patients with juvenile idiopathic arthritis: a cross-sectional study. Ann Rheum Dis.

[7] Fadini GP, Losordo D, Dimmeler S (2012). Critical reevaluation of endothelial progenitor cell phenotypes for therapeutic and diagnostic use. Circ Res.

[8] Pozzoli O, Vella P, Iaffaldano G, Parente V, Devanna P, Lacovich M (2011). Endothelial fate and angiogenic properties of human CD34+ progenitor cells in zebrafish. Arterioscler Thromb Vasc Biol.

[9] Westerweel PE, Verhaar MC (2009). Endothelial progenitor cell dysfunction in rheumatic disease. Nat Rev Rheumatol.

[10] van Zonneveld AJ, de Boer HC, van der Veer EP, Rabelink TJ (2010). Inflammation, vascular injury and repair in rheumatoid arthritis. Ann Rheum Dis.

[11] Haque S, Alexander MY, Bruce IN (2012). Endothelial progenitor cells: a new player in lupus?. Arthritis Res Ther.

[12] Distler JH, Beyer C, Schett G, Luscher TF, Gay S, Distler O (2009). Endothelial progenitor cells: novel players in the pathogenesis of rheumatic diseases. Arthritis Rheum.

[13] Fadini GP, Tognon S, Rodriguez L, Boscaro E, Baesso I, Avogaro A (2009). Low levels of endothelial progenitor cells correlate with disease duration and activity in patients with Behcet’s disease. Clin Exp Rheumatol.

[14] Porta C, Caporali R, Epis O, Ramaioli I, Invernizzi R, Rovati B (2004). Impaired bone marrow hematopoietic progenitor cell function in rheumatoid arthritis patients candidated to autologous hematopoietic stem cell transplantation. Bone Marrow Transplant.

[15] Fadini GP, Albiero M, Seeger F, Poncina N, Menegazzo L, Angelini A (2013). Stem cell compartmentalization in diabetes and high cardiovascular risk reveals the role of DPP-4 in diabetic stem cell mobilopathy. Basic Res Cardiol.

[16] Fadini GP, Albiero M, Boscaro E, Cappellari R, Marescotti M, Poncina N (2013). Diabetes impairs stem cell and proangiogenic cell mobilization in humans. Diabetes Care.

[17] Papadaki HA, Kritikos HD, Gemetzi C, Koutala H, Marsh JC, Boumpas DT (2002). Bone marrow progenitor cell reserve and function and stromal cell function are defective in rheumatoid arthritis: evidence for a tumor necrosis factor alpha-mediated effect. Blood.

[18] Ruger B, Giurea A, Wanivenhaus AH, Zehetgruber H, Hollemann D, Yanagida G (2004). Endothelial precursor cells in the synovial tissue of patients with rheumatoid arthritis and osteoarthritis. Arthritis Rheum.

[19] Silverman MD, Haas CS, Rad AM, Arbab AS, Koch AE (2007). The role of vascular cell adhesion molecule 1/ very late activation antigen 4 in endothelial progenitor cell recruitment to rheumatoid arthritis synovium. Arthritis Rheum.

[20] Grisar J, Aletaha D, Steiner CW, Kapral T, Steiner S, Saemann M (2007). Endothelial progenitor cells in active rheumatoid arthritis: effects of tumour necrosis factor and glucocorticoid therapy. Ann Rheum Dis.

[21] Li Calzi S, Neu MB, Shaw LC, Kielczewski JL, Moldovan NI, Grant MB (2010). EPCs and pathological angiogenesis: when good cells go bad. Microvasc Res.

[22] Fadini GP, Maruyama S, Ozaki T, Taguchi A, Meigs J, Dimmeler S (2010). Circulating progenitor cell count for cardiovascular risk stratification: a pooled analysis. PLoS One.

[23] McEntegart A, Capell HA, Creran D, Rumley A, Woodward M, Lowe GD (2001). Cardiovascular risk factors, including thrombotic variables, in a population with rheumatoid arthritis. Rheumatology (Oxford).

[24] Berenson GS, Wattigney WA, Tracy RE, Newman WP, Srinivasan SR, Webber LS (1992). Atherosclerosis of the aorta and coronary arteries and cardiovascular risk factors in persons aged 6 to 30 years and studied at necropsy (The Bogalusa Heart Study). Am J Cardiol.

[25] Chen CY, Tsao CH, Ou LS, Yang MH, Kuo ML, Huang JL (2002). Comparison of soluble adhesion molecules in juvenile idiopathic arthritis between the active and remission stages. Ann Rheum Dis.

[26] Coulson EJ, Ng WF, Goff I, Foster HE (2013). Cardiovascular risk in juvenile idiopathic arthritis. Rheumatology (Oxford).

[27] Vlahos AP, Theocharis P, Bechlioulis A, Naka KK, Vakalis K, Papamichael ND (2011). Changes in vascular function and structure in juvenile idiopathic arthritis. Arthritis Care Res (Hoboken).

[28] Seeger FH, Haendeler J, Walter DH, Rochwalsky U, Reinhold J, Urbich C (2005). p38 mitogen-activated protein kinase downregulates endothelial progenitor cells. Circulation.

[29] Spinelli FR, Metere A, Barbati C, Pierdominici M, Iannuccelli C, Lucchino B (2013). Effect of Therapeutic Inhibition of TNF on Circulating Endothelial Progenitor Cells in Patients with Rheumatoid Arthritis. Mediators Inflamm.

[30] Distler JH, Allanore Y, Avouac J, Giacomelli R, Guiducci S, Moritz F (2009). EULAR Scleroderma Trials and Research group statement and recommendations on endothelial precursor cells. Ann Rheum Dis.

